# Supplementary material for: Direct and indirect pathways linking the Lon protease to motility behaviors in the pathogen Pseudomonas aeruginosa
Source: PLoS Pathog. 2025 Jun 25;21(6):e1013288. doi: 10.1371/journal.ppat.1013288 (PMC12221181; doi:10.1371/journal.ppat.1013288)
Supplement: S1 Table — (PDF) [file ppat.1013288.s007.pdf]

S1 Table. Strains used in the study.

Pseudomonas aeruginosa strains				
**Name**	**Description**	**Genotype**	**Marker**	**Reference**
8277	Clone C strain urine isolate, C2	Wild type	-	[1]
KJ1227	Clone C wild type strain transformed with empty vector pJN105	WT + pJN105	gent^R	-
KJ1228	Clone C wild type strain transformed with vector pJN105 containing *lon*	WT + pAK009	gent^R	-
H103	PAO1 WT	Wild-type *P. aeruginosa* PAO1 strain H103	-	Provided by Robert Hancock, University of British Columbia
KJ1230	PAO1 wild type strain transformed with empty vector pJN105	WT + pJN105	gent^R	-
KJ1231	PAO1 wild type strain transformed with vector pJN105 containing *lon*	WT + pAK009	gent^R	-
H1105	*lon*- transposon insertion mutant	PAO1 mini-Tn*5*–*luxCDABE*::*lon*; 74_D9, frameshift mutation in *pilI*	tet^R	[2]
KJ1233	*lon*- wild type strain transformed with empty vector pJN105	PAO1 mini-Tn*5*–*luxCDABE*::*lon*; 74_D9, frameshift mutation in *pilI* + pJN105	gent^R	-
KJ1234	*lon*- strain transformed with vector pJN105 containing *lon*	PAO1 mini-Tn*5*–*luxCDABE*::*lon*; 74_D9, frameshift mutation in *pilI* + pAK009	gent^R	-
KJ1300	PAO1 wild type strain transformed with vector pJN105 containing *pilI*	WT + pAK033	gent^R	-
KJ1301	*lon*- strain transformed with vector pJN105 containing *pilI*	PAO1 mini-Tn*5*–*luxCDABE*::*lon*; 74_D9, frameshift mutation in *pilI* + pAK033	gent^R	-
KJ1302	PAO1 wild type strain transformed with vector pJN105 containing N-FLAG-*sulA*	WT + pAK029	gent^R	-
KJ1303	PAO1 wild type strain transformed with vector pJN105 containing N-FLAG-*sulA*^{H127L}	WT + pAK030	gent^R	-
KJ1304	PAO1 wild type strain transformed with vector pJN105 containing N-FLAG-*sulA*^{D109V}	WT + pAK031	gent^R	-
KJ1305	*lon*- strain transformed with vector pJN105 containing N-FLAG-*sulA*^{fs}	WT + pAK032	gent^R	-

Escherichia coli **strains**				
**Name**	**Description**	**Genotype**	**Marker**	**Reference**
-	DH5α	General cloning strain	-	Invitrogen
BL21-SI/ pCodonPlus	Salt-inducible BL21(DE3) strain for protein expression	Protein expression strain	$chlor^R$	Provided by Claes Andréasson, Stockholm University

References

1. Dinesh SD, Grundmann H, Pitt TL, Römling U. European-wide distribution of Pseudomonas aeruginosa clone C. Clinical Microbiology and Infection. 2003;9: 1228–1233. doi:10.1111/j.1469-0691.2003.00793.x

2. Lewenza S, Falsafi RK, Winsor G, Gooderham WJ, McPhee JB, Brinkman FSL, et al. Construction of a mini-Tn *5-luxCDABE* mutant library in *Pseudomonas aeruginosa* PAO1: A tool for identifying differentially regulated genes. Genome Res. 2005;15: 583–589. doi:10.1101/gr.3513905
