# Supplementary material for: Direct and indirect pathways linking the Lon protease to motility behaviors in the pathogen Pseudomonas aeruginosa
Source: PLoS Pathog. 2025 Jun 25;21(6):e1013288. doi: 10.1371/journal.ppat.1013288 (PMC12221181; doi:10.1371/journal.ppat.1013288)
Supplement: S2 Table — (PDF) [file ppat.1013288.s008.pdf]

S2 Table. Plasmids used in this study**.**

**Name**	**Description**	**Marker**	**Reference**
pJN105	Broad-host range vector with L-arabinose-inducible *araBAD* promoter; pBBR1ori	$gent^R$	[1]
pAK009	pJN105 containing *lon*	$gent^R$	This study
pSUMO-YHRC	Plasmid for protein expression using P_{T7} with an N-terminal 6xHis-SUMO tag; RRID: Addgene_54336	kan^R	[2]
pAK010	pSUMO-YHRC containing *6xHis-SUMO-lon*	kan^R	This study
pAK011	pSUMO-YHRC containing *6xHis-SUMO-sulA*	kan^R	This study
pAK012	pSUMO-YHRC containing *6xHis-SUMO-fliG*	kan^R	This study
pAK013	pSUMO-YHRC containing *6xHis-SUMO-flgE*	kan^R	This study
pAK014	pSUMO-YHRC containing *6xHis-SUMO-fliA*	kan^R	This study
pAK015	pSUMO-YHRC containing *6xHis-SUMO-rpoN*	kan^R	This study
pAK016	pSUMO-YHRC containing *6xHis-SUMO-amrZ*	kan^R	This study
pAK017	pSUMO-YHRC containing *6xHis-SUMO-fliS*	kan^R	This study
pAK018	pSUMO-YHRC containing *6xHis-SUMO-fliS2*	kan^R	This study
pAK019	pSUMO-YHRC containing *6xHis-SUMO-ibpA*	kan^R	This study
pAK020	pSUMO-YHRC containing *6xHis-SUMO-speH*	kan^R	This study
pAK021	pSUMO-YHRC containing *6xHis-SUMO-bioB*	kan^R	This study
pAK022	pSUMO-YHRC containing *6xHis-SUMO-rlmN*	kan^R	This study
pAK029	pJN105 containing N-ter FLAG-*sulA*	$gent^R$	This study
pAK030	pJN105 containing N-ter FLAG-$sulA^{H127L}$	$gent^R$	This study
pAK031	pJN105 containing N-ter FLAG-$sulA^{D109V}$	$gent^R$	This study
pAK032	pJN105 containing N-ter FLAG-$sulA^{fs}$	$gent^R$	This study
pAK033	pJN105 containing *pilI*	$gent^R$	This study

References

1. Newman JR, Fuqua C. Broad-host-range expression vectors that carry the l-arabinose-inducible Escherichia coli araBAD promoter and the araC regulator. Gene. 1999;227: 197–203. doi:10.1016/S0378-1119(98)00601-5

2. Holmberg MA, Gowda NKC, Andréasson C. A versatile bacterial expression vector designed for single-step cloning of multiple DNA fragments using homologous recombination. Protein Expr Purif. 2014;98: 38–45. doi:10.1016/j.pep.2014.03.002
