# Supplementary material for: Direct and indirect pathways linking the Lon protease to motility behaviors in the pathogen Pseudomonas aeruginosa
Source: PLoS Pathog. 2025 Jun 25;21(6):e1013288. doi: 10.1371/journal.ppat.1013288 (PMC12221181; doi:10.1371/journal.ppat.1013288)
Supplement: S3 Table — (PDF) [file ppat.1013288.s009.pdf]

S3 Table. Primers used in this study.

Name	Sequence (5'-3')	Description	Reference
OAK029	CCATACCCGTTTTTTTGGGCTAGCATGAAAACACTCGTCGAATTGC	pJN105 NheI *lon*OE cloning for	This study
OAK030	CTAGTGGATCCCCCGGGCTGCAGCTAATGCGTGCTAATTCGCTC	pJN105 PstI *lon*OE cloning rev	This study
OAK031	TAATACGACTCACTATAGGG	T7 promoter for	common primer
OAK032	GCTAGTTATTGCTCAGCGG	T7 terminator rev	common primer
OAK033	AGGGTTTTCCCAGTCACGACGTT	M13 rev	common primer
OAK034	GAGCGGATAACAATTTCACACAG	M13 for	common primer
OAK035	CGGCGTCACACTTTGCTATG	Sequencing *lon*OE insert in pJN105 for	This study
OAK036	CCTGGACCTGCTTCGGAATC	Sequencing *lon*OE insert in pJN105 rev	This study
OAK037	CACAGAGAACAGATTGGTGGGATGAAAACACTCGTCGAATTGC	*lon* insert for pSUMO-YHRC for	This study
OAK038	GACGGAGCTCGAATTCGGATCCTAATGCGTGCTAATTCGCTC	*lon* insert for pSUMO-YHRC rev	This study
OAK039	CACAGAGAACAGATTGGTGGGATGCAGACCTCCCACTCGCTG	*sulA* insert for pSUMO-YHRC for	This study
OAK040	GACGGAGCTCGAATTCGGATCTCAACCCAGACGAATATTCAG	*sulA* insert for pSUMO-YHRC rev	This study
OAK091	CACAGAGAACAGATTGGTGGGATGAGTGAGAATCGTCTCGCCG	*fliG* insert for pSUMO-YHRC for	This study
OAK092	GACGGAGCTCGAATTCGGATCTCAGATCATCTCCTCGCCACCCTTG	*fliG* insert for pSUMO-YHRC rev	This study
OAK134	CACAGAGAACAGATTGGTGGGATGAGTTTCAACATCGGCCTGAGCGGCATCCAGGC	*flgE* insert for pSUMO-YHRC for	This study
OAK135	GAGCTCGAATTCGGATCTCAGCGCAGGTTGATGATGGTCTGGGTCACCGCATCCTCGGTC	*flgE* insert for pSUMO-YHRC rev	This study
OAK136	CACAGAGAACAGATTGGTGGGATGACAGCGGCCTCTGGAGTGCGTATGTATAGC	*fliA* insert for pSUMO-YHRC for	This study
OAK137	GAGCTCGAATTCGGATCTCAGGCCGACCGCCAATCGGCCAGGCGCGCGCGCAAAC	*fliA* insert for pSUMO-YHRC rev	This study
OAK138	CACAGAGAACAGATTGGTGGGATGAAACCATCGCTAGTCCTCAAGATGGGCCAGC	*rpoN* insert for pSUMO-YHRC for	This study
OAK139	GAGCTCGAATTCGGATCTCACACCAGTCGCTTGCGCTCGCTCGAAGGGGC	*rpoN* insert for pSUMO-YHRC rev	This study
OAK140	CACAGAGAACAGATTGGTGGGATGCGCCCACTGAAACAGGCAACTCCTACCTAC	*amrZ* insert for pSUMO-YHRC for	This study
OAK141	GAGCTCGAATTCGGATCTCAGGCCTGGGCCAGCTCCGCATCGTGTGCGATC	*amrZ* insert for pSUMO-YHRC rev	This study

Name	Sequence (5'-3')	Description	Reference
OAK175	CACAGAGAACAGATTGGTGGGATGAACGCAATGGCAGCCATGCGGCAATAC	*fliS* insert for pSUMO-YHRC for	This study
OAK176	GAGCTCGAATTCGGATCTCAGGGGGCAATCGCATCCCAACCGGATTTGATG	*fliS* insert for pSUMO-YHRC rev	This study
OAK177	CACAGAGAACAGATTGGTGGGATGTCGCGTCCTATCGATACCTACCGGCAG	*fliS2* insert for pSUMO-YHRC for	This study
OAK178	GGAGCTCGAATTCGGATCTTAGCGCCGTTCGCTGTCTTCGCCCTGCGCTTCG	*fliS2* insert for pSUMO-YHRC rev	This study
OAK089	CACAGAGAACAGATTGGTGGGATGAGCAACGCTTTTTCCCTCG	*ibpA* insert for pSUMO-YHRC for	This study
OAK090	GACGGAGCTCGAATTCGGATCTTACTGGTTGTCCAGTGCCG	*ibpA* insert for pSUMO-YHRC rev	This study
OAK109	CACAGAGAACAGATTGGTGGGATGGCAATTCAACCGTTGCGACTCGATCCG	*speH* insert for pSUMO-YHRC for	This study
OAK110	GACGGAGCTCGAATTCGGATCTCAGGCCACCCCCGTGCCCTGGCCCAGCGC	*speH* insert for pSUMO-YHRC rev	This study
OAK093	CACAGAGAACAGATTGGTGGGATGAGTGCAACCGCTTCCGTCGCCACCCG	*bioB* insert for pSUMO-YHRC for	This study
OAK094	GACGGAGCTCGAATTCGGATCTCAGGCGGAAGCGGCGTTATAGAACAGC	*bioB* insert for pSUMO-YHRC rev	This study
OAK111	CACAGAGAACAGATTGGTGGGATGACAACGACCACCGCCGGCAAGGTGAAC	*rlmN* insert for pSUMO-YHRC for	This study
OAK112	GACGGAGCTCGAATTCGGATCTCAATTTCGATTCGCCGCCGACTCGGACTC	*rlmN* insert for pSUMO-YHRC rev	This study
OAK225	CCATACCCGTTTTTTTGGGCTAGCATGTCGGACGTTCAGACCCCCTTC	pJN105 NheI *pilI*OE cloning for	This study
OAK226	CTAGTGGATCCCCCGGGCTGCAGTTATACGGCGACGTCGAGGAAGC	pJN105 PstI *pilI*OE cloning rev	This study
OAK231	GACTACAAGGACGACGACGACAAGCATATGATGCAGACCTCCCACTCGC	*sulA or sulA* mutant N-ter FLAG-tag insert for pJN105 for	This study
OAK232	CTCCACCGCGGTGGCGGCCGCTCTAGAACTAGTttaCATATGTCAACCCAGACGAATATTC	*sulA or sulA* mutant N-ter FLAG-tag insert for pJN105	This study
oMJF34	CCCACCAATCTGTTCTCTGTG	Cloning pSUMO-YHRC fragment 1	[1]
oMJF36	CATGCATCATCAGGAGTACGG	Cloning pSUMO-YHRC fragment 1	
oMJF37	GATCCGAATTCGAGCTCC	Cloning pSUMO-YHRC fragment 2	
oMJF38	GAATTTATGCCTCTTCCGACC	Cloning pSUMO-YHRC fragment 2	

References

1. Omnus DJ, Fink MJ, Szwedo K, Jonas K. The Lon protease temporally restricts polar cell differentiation events during the Caulobacter cell cycle. eLife. 2021;10: e73875. doi:10.7554/eLife.73875
