# Supplementary material for: Direct and indirect pathways linking the Lon protease to motility behaviors in the pathogen Pseudomonas aeruginosa
Source: PLoS Pathog. 2025 Jun 25;21(6):e1013288. doi: 10.1371/journal.ppat.1013288 (PMC12221181; doi:10.1371/journal.ppat.1013288)
Supplement: Attachment — Submitted filename: Response to reviewers.pdf [file ppat.1013288.s013.pdf]

Point-to-point response to reviewers (manuscript PPATHOGENS-D-25-00035)

We thank the reviewers as well as the editor for the time they have invested to assess our work and provide these helpful and constructive suggestions. We have carefully considered their comments and have addressed each point either experimentally and/or by modifying the manuscript text to increase the clarity of our work. Please find our responses below, with the original points appearing in black and our replies in blue.

Additional Editor Comments:

Thanks for submitting the manuscript. The reviewers identified these issues that should be addressed:

1. There is concern whether the use of spectinomycin leads to complete or partial inhibition. Determining this experimentally, such as a pulse chase, under the conditions used in this study would be good.
This is indeed a valid point. We have chosen spectinomycin based on previous studies by the Römling lab and other groups, in which it was used for *in vivo* protein degradation assays in *P. aeruginosa*. Before conducting our proteomics experiments, we have tested the effect of a range of spectinomycin concentrations on *P. aeruginosa* growth. Based on this experiment, a concentration of 1 mg/ml was found to be needed to strongly inhibit culture growth and we decided to use this concentration for our *in vivo* stability assays. While the observed growth reduction at this concentration indicates that protein synthesis is strongly inhibited, we cannot completely rule out that protein synthesis continues at a low rate or of a fraction of proteins. Hence, we have modified our text to open for this possibility. We have also added the data showing the effect of different spectinomycin concentrations on growth as a new supplementary figure (Fig. S1B). The editor's suggestion to experimentally test the efficiency of spectinomycin by a pulse-chase experiment is in principle a good idea, however due to the lack of specific antibodies for known Lon substrates in *P. aeruginosa* we were not able to conduct this type of analysis.

2. Statistics should be provided for Figure 3.
We have included statistics for the data presented in Fig. 3. This shows that degradation of FliG and FlgE is significantly affected in the *lon-* mutant. The Lon-dependent effect on RpoN and FliS degradation was milder and according to the statistical tests not significant. We have modified the text to describe the protein stability data more accurately and have also changed the subtitle of this section to a title that better reflects our findings.

3. Determine the whether the protein level of the suppressor alleles of sulA
In order to monitor native protein levels of SulA by Western blotting, we rely on SulA-specific antibodies. We have ordered antibodies specific for *E. coli* SulA, which unfortunately do not work for *P. aeruginosa* SulA. To still be able to assess how the different suppressor mutations affect SulA protein levels, we have expressed wild-type SulA and the mutant SulA variants (H127L, D109V and frameshift) with an N-terminal 3xFLAG-tag from a P_{BAD}-promoter in the wild-type background and measured their levels using an anti-FLAG antibody. This analysis showed that the mutant proteins H127L and D109V were robustly expressed, albeit at lower levels compared to wild-type SulA, while the SulA variant with the frameshift mutation was undetectable, likely due to protein instability.
In addition to this Western blot analysis, we have also assessed cell morphology and soft-agar motility of the strains expressing SulA and the different SulA mutants. While SulA expression resulted in strong cell filamentation, as expected, expression of the SulA mutants resulted either in no filamentation (H127L and frameshift) or in case of the D109V mutant in moderate cell filamentation. Importantly, the reduced cell filamentation of the SulA mutants correlated with improved motility. Our new data are consistent with our conclusion that the suppressor mutations result in a complete or partial loss of SulA function, respectively. Given that the H127L and D109V mutants are still robustly expressed, it is likley that their interaction with FtsZ is disrupted, as proposed in our discussion. Furthermore, they show that SulA-induced cell filamentation correlates with reduced motility in soft-agar.
We have incorporated these new data in Fig. 5 and Fig. S5.

4. The inability to complement lonA from a plasmid for twitching and biofilm is curious, but no

explanation is provided. Can different amount of inducer be tested to determine if the inability to complement is due to the amount of LonA made from the plasmid?
We have obtained similar biofilm formation and twitching data with high and low / leaky P_{ara}-*lon* expression (see also Fig. S3 for the different Lon expression levels), indicating that the observed lack of complementation is not a matter of Lon expression levels.
Instead, we explain the inability to complement the twitching and biofilm phenotypes with the presence of the frameshift mutation in *pilI* in the *lon-* strain that we discovered after re-sequencing the genomes of the suppressor mutants along with their parental *lon-* strain. PilI is a component of the type IV pilus, hence a mutation of the corresponding gene is expected to cause changes in adhesion, biofilm formation and twitching.
Based on this comment by the editor as well as comments from the two reviewers (comment #4 by reviewer 2 and major comment #4 by reviewer 1), we decided to experimentally test if the *pilI* background mutation causes the biofilm and twitching phenotypes of the *lon-* strain. For this, we expressed a plasmid-borne wild-type copy of *pilI* from a P_{BAD}- promoter in the *lon-* strain. Indeed, *pilI* expression in the *lon-* strain completely restored biofilm formation and significantly increased twitching motility upon ectopic expression of *pilI*, demonstrating that the biofilm and twitching phenotypes are largely due to the frameshift mutation in *pilI*. This is an important finding as it challenges the previously reported roles of Lon in biofilm formation and twitching motility and also emphasizes the importance of checking the genetic backgrounds of *lon-* strains. Hence, we have added these data together with a more detailed description of the *pilI* mutation (new figure Fig. 6) and describe this new finding in the text.

Journal Requirements:

1) Please ensure that the CRediT author contributions listed for every co-author are completed accurately and in full.
   At this stage, the following Authors/Authors require contributions: Aswathy Kallazhi, Anamika Rahman, and Ute Römling. Please ensure that the full contributions of each author are acknowledged in the "Add/Edit/Remove Authors" section of our submission form.
   The list of CRediT author contributions may be found here: https://journals.plos.org/plospathogens/s/authorship#loc-author-contributions

CRediT author contributions have been added.

2) We ask that a manuscript source file is provided at Revision. Please upload your manuscript file as a .doc, .docx, .rtf or .tex. If you are providing a .tex file, please upload it under the item type u2018LaTeX Source Fileu2019 and leave your .pdf version as the item type u2018Manuscriptu2019.

Manuscript is provided as .docx file.

3) We do not publish any copyright or trademark symbols that usually accompany proprietary names, eg ©, ®, or TM (e.g. next to drug or reagent names). Therefore please remove all instances of trademark/copyright symbols throughout the text, including:
   - ® on pages: 25 and 26.
   - TM on pages: 25 and 26.

Trademark/copyright symbols have been removed

4) Please upload all main figures as separate Figure files in .tif or .eps format. For more information about how to convert and format your figure files please see our guidelines: https://journals.plos.org/plospathogens/s/figures

Done.

5) We have noticed that you have uploaded Supporting Information files, but you have not included a list of legends. Please add a full list of legends for your Supporting Information files after the references list.

We have included a list of legends for the Supporting Information files.

6) Some material included in your submission may be copyrighted. According to PLOSu2019s copyright policy, authors who use figures or other material (e.g., graphics, clipart, maps) from another author or copyright holder must demonstrate or obtain permission to publish this material under the Creative Commons Attribution 4.0 International (CC BY 4.0) License used by PLOS journals. Please closely review the details of PLOSu2019s copyright requirements here: PLOS Licenses and Copyright. If you need to request permissions from a copyright holder, you may use PLOS's Copyright Content Permission form.

   Please respond directly to this email and provide any known details concerning your material's license terms and permissions required for reuse, even if you have not yet obtained copyright permissions or are unsure of your material's copyright compatibility. Once you have responded and addressed all other outstanding technical requirements, you may resubmit your manuscript within Editorial Manager.

   Potential Copyright Issues:

   - Please confirm that you are the photographer of Figure 4D, or provide written permission from the photographer to publish the photo under our CC BY 4.0 license.

We are the photographer of Figure 4D.

Reviewers' Comments:

Reviewer's Responses to Questions

Part I - Summary
Please use this section to discuss strengths/weaknesses of study, novelty/significance, general execution and scholarship.

Reviewer #1: The ATP-dependent Lon protease plays vital roles in protein control across all life domains. Lon's absence causes multiple defects in motility, virulence, antibiotic tolerance, and biofilm formation in a pathogenic bacterium Pseudomonas aeruginosa. Through proteome analysis of Lon overexpression, authors identified new Lon substrate proteins, particularly those involved in motility and cell division. They confirmed several new substrates through in vitro protein degradation assay, including proteins crucial for flagella and pilus-based movement. Additionally, they discovered that Lon's degradation of SulA, a cell division inhibitor, is essential for proper cell division and motility. This work expands our understanding of how Lon protease regulation contributes to P. aeruginosa pathogenicity. While these findings are intriguing, the authors note that the manuscript requires further revisions in certain areas.

Reviewer #2: In their manuscript "Direct and indirect pathways linking the Lon protease to motility behaviors in the pathogen Pseudomonas aeruginosa", Kallazhi and colleagues describe the identification and characterisation of substrates of the Lon protease in Pseudomonas aeruginosa. They first perform quantitative proteomics to compare a strain overexpressing Lon to the matched WT strain from P. aeruginosa clone C, before and after inhibiting new protein synthesis with spectinomycin. This identifies 64 candidate substrates that satisfy 3 different analysis criteria. The authors then show that Lon can degrade nine of these proteins in vitro (two candidate substrates that were tested did not show in vitro degradation). Next, they investigate whether the steady state levels or stabilities of these substrates are affected by lon disruption in a different strain (PAO1) and find that some of them are stabilised in the lon- strain, increasing the chances that they are bona fide substrates. Finally, they investigate phenotypes predicted to be affected by the new Lon substrates and find that motility, growth, biofilm production, and pigment production do in fact seem to be affected by Lon. They show that

several of the phenotypes can be reverted by sulA suppressor mutations, but also that their lon- strain carries a background mutation in pilI, which likely impacts twitching motility and biofilm formation. Overall, these findings contribute to understanding of the roles of the Lon protease in P. aeruginosa regulation and physiology. The network of regulators in which Lon is embedded is complex, with many examples of crosstalk at multiple regulatory levels, and the observations presented here are interesting and useful. In particular, the observation that sulA suppressor mutations revert many lon- phenotypes is important. However, several complexities impact the interpretation of the data, and in some places, clarity could be improved.

We thank both reviewers for their positive comments and are pleased that they consider our work to be interesting.

Some suggestions for this are listed below:

1. There is good evidence at least in E. coli (PMID: 39003262), and to a lesser extent in P. aeruginosa (PMID: 32029587), that the AAA+ ATPase dependent protease families (Lon, ClpXP, HslUV, and FtsH) have some degree of redundancy or partially overlapping substrates. The abilities of these proteases to degrade overlapping subsets of substrate proteins seems like an important explanatory point for reconciling the results observed in the lon overexpression experiment with the results in the lon disruption experiment. This point is very briefly mentioned in the discussion, but it seems worth bringing it up earlier, and the assertion in the introduction that each protease "degrades a distinct panel of substrates" should be clarified to accommodate this evidence that their substrates and functions are overlapping.

This is a good suggestion. We have added some more background in the introduction to better explain that the substrate pools of AAA+ proteases can overlap to varying degrees and under certain conditions suggesting some redundancy. We have added the suggested citations to support these statements.

2. The use of spectinomycin to inhibit new protein synthesis so that degradation rates could be assessed at the proteome level is useful for improving sensitivity to detect Lon impacts. However, spectinomycin has been described as an incomplete inhibitor of translation, that only slows but does not completely stop translocation. This does not necessarily invalidate it for use in this experiment - slowing the rate of new protein synthesis still can impart a relative increase on the importance of protein degradation in determining the levels of proteins. However, mentioning the possibility of some ongoing protein synthesis could potentially help explain some issues in the proteomics data: 1) substantial increases in abundance over time during the translation inhibition is observed for some proteins; and 2) the fold change from time 0 to time 30 is very similar to the fold change from 0 to time 60 for several of the candidate Lon substrates. If slow ongoing synthesis favoured some proteins over others, and/or if slow new synthesis could more or less keep pace with degradation after the initial decrease in new synthesis established a lower steady state level, these observations could be explained. Alternatively, artefacts of normalisation in the context of generally decreasing protein abundance during spectinomycin treatment could contribute to these issues. It would be good for the authors to comment on these patterns and the explanation(s) they favour.

The reviewer raises an important point that may explain some of our data. As outlined in our response to the editor, we have used a spectinomycin concentration of 1 mg/ml for our *in vivo* degradation assays at which growth is strongly inhibited. While the growth reduction indicates an efficient inhibition of protein synthesis, we agree that protein synthesis might continue at a low rate or of some specific proteins. This could indeed explain that some proteins in our datasets increase in abundance after spectinomycin addition as well as the rather mild changes between the 30 min and 60 min time points. Based on the reviewer's suggestion, we have added a sentence to mention that the protein synthesis shut-off might not be complete and how this might affect our data. Additionally, we have added Supplementary Fig. 1B to show that the chosen spectinomycin concentration strongly inhibits culture growth.

3. The list of newly identified Lon substrates is interesting for many reasons. One reason is that several of them are known to play roles as chaperones or binding partners that contribute to protein stabilisation, folding, and degradation, raising the possibility that there could be complex feedback effects. IbpA is one example - it is a chaperone of unfolded proteins that is upregulated by heat shock and starvation. Could disruption of proteostasis through Lon dysregulation affect the availability of unfolded proteins

for IbpA to bind, and thus affect its own availability for degradation? The flagellar synthesis cascade is another example of this: FliS is a chaperone for FliC secretion; FlgE is required for FlgM secretion, and FlgM is the chaperone/anti-sigma factor for FliA, which is also the sigma factor driving FliC expression. Several of these proteins have intrinsically disordered regions on their own. Does Lon do a job of degrading any of these proteins that are in excess stoichiometrically and not bound to their proper binding partners? Such a homeostasis-promoting role could explain the lack of disruption to steady state levels in the lon- strain and the ability of the sulA suppressor mutation to completely alleviate the lon- strain's motility defect.

These are a number of interesting points. It is an interesting idea that Lon degradation of some of the substrates might have a homeostasis-promoting role, rather than a regulatory purpose. We agree that Lon might contribute to the degradation of flagella proteins in *P. aeruginosa* that are in stoichiometric excess and not bound to their binding partners and we included a sentence in the discussion to suggest this possibility. We also agree that the relationship between Lon and IbpA is interesting and might indicate a more complex interplay between these factors in the context of the proteostasis network. While we chose in our study to focus on other substrates and to explain previously reported *lon-* phenotypes, it will be interesting to dissect the functional relationship between Lon and IbpA as well as other substrates in future research.

4. The discovery of a background mutation in the lon- strain which might explain the biofilm and twitching phenotypes seems like an important point. Is there selection in lon- strains for type IV pilus mutations? Do you think that previously reported biofilm and twitching phenotypes are also likely to be due to background mutations? Mutations to type IV pilus genes are very common in clinical isolates and seem to confer advantages in a variety of infection-mimicking conditions; perhaps they can also alleviate stress imposed by lon mutation.

We agree that the *pilI* mutation in the *lon-* mutant background was an important finding that will also affect the interpretation of previously reported biofilm and twitching phenotypes of the *lon-* mutant. Our new data show that ectopically expressing wild-type *pilI* in the *lon-* background fully or partially complements the biofilm and twitching phenotypes of this strain, respectively (see our response to comment # 4 by the editor). This clearly demonstrates that these phenotypes are strongly affected by the *pilI* mutation and questions the previously reported role of Lon in biofilm formation. It is indeed interesting to consider why the *pilI* mutation might have arosen in the *lon-* background and if there is selective pressure for losing *pilI* function in a *lon-* mutant.

5. Since it is mentioned in the introduction as a previously identified substrate of Lon in P. aeruginosa, it might be worth commenting on the status of Hfq in these proteomics data sets.

SulA and LasI were not detected in our proteomics experiment. Hfq was detected and showed mildly reduced leves in the *lon* overexpression strain, but did not satisfy the other criteria. We have added this information to the text.

Part II – Major Issues: Key Experiments Required for Acceptance
Please use this section to detail the key new experiments or modifications of existing experiments that should be absolutely required to validate study conclusions.

Generally, there should be no more than 3 such required experiments or major modifications for a "Major Revision" recommendation. If more than 3 experiments are necessary to validate the study conclusions, then you are encouraged to recommend "Reject".

Reviewer #1: Major concerns

1. Although this manuscript identifies SulA degradation by Lon protease as a key finding, similar mechanisms have already been characterized in other bacterial species. Therefore, the manuscript needs to clearly highlight the novel aspects specific to P. aeruginosa and demonstrate how they differ from previously established mechanisms in other bacteria.

The novelty of our work lies in demonstrating the importance of keeping SulA levels low by Lon in *P. aeruginosa* and how an inability to do so (*i.e.*, in the *lon*- mutant) affects cell division and as a consequence of this motility and other pathogenicity-related phenotypes. Additionally, there are several other interesting aspects of our findings regarding Lon and SulA. For example, the suppressor mutations in SulA reveal residues critical for SulA function on cell division, likely through its interaction with FtsZ. It is also interesting that the SulA proteins from *E. coli* and *P. aeruginosa* show sequence divergence in their C-termini, indicating that the interaction between Lon and SulA is conserved independent of their specific degron sequences.

In addition to dissecting the physiological importance of Lon-dependent degradation of SulA in *P. aeruginosa*, our study identifies a large set of other interesting Lon substrate proteins and also explains previously reported biofilm and twitching phenotypes of the *lon*- strain with a background mutation in *pilI*. Together, these are important findings to clarify the precise role of Lon in *Pseudomonas* physiology.

In response to this comment, we have modified the discussion of our paper to emphasize even more the novel aspects of our work, particularly with regard to SulA.

2. Line 217-218: to verify the mechanism of compensated phenotypes, authors should either analyze transcriptional regulation using specific transcriptional regulators, or alternatively, treat samples with rifampicin (a transcriptional inhibitor) to exclude the possibility of transcriptional regulation.

We have changed the text of this section to better explain the data presented in Fig. 3. While compensatory transcriptional effects might explain the behaviors of FlgE and FliG, degradation by other proteases or protective protein interactions might explain the degradation behaviors of the other substrates. Establishing the precise reason for the degradation of each of these proteins, would go too far in our eyes, and might in some cases also be technically difficult (as discussed in the discussion). Hence, we decided to focus in the remaining part of our paper on explaining previously reported phenotypes of *lon*- cells relevant to *Pseudomonas* pathogenicity. However, in response to this comment as well as suggestions by the editor, we have changed the text describing Fig. 3 to more accurately describe the data. Furthermore, we have weakened our statements regarding compensatory transcriptional regulation.

3. Figure 3: The authors suggested increased stability of FliG, FliS, FlgE, and RpoN in the lon mutant strain compared to wild-type. However, this comparison lacks statistical analysis to validate these differences. Furthermore, statistical analyses are missing from multiple figures throughout the manuscript, which is necessary to support the significance of the reported findings.

We have added the requested statistical tests. This analysis confirmed that degradation of FliG and FlgE was significantly slower in the *lon*- mutant compared to the wild type. On the other hand, the effect of Lon on FliS and RpoN was not significant. We have modified our text to describe our data more carefully. Additionally, we have rephrased the subtitle of this section to better reflect our actual data. We have also double-checked that the results of statistical tests are included in the other figures, when relevant.

4. Line 250-252: The twitching and biofilm phenotypes fail to recover in the lon mutant strain, unlike other phenotypes. The authors should address this discrepancy either by testing alternative plasmid systems, or if this approach is not feasible, provide a detailed explanation for why these two phenotypes differ from the others in their recovery pattern.

See also comment # 4 to the editor. Our new data demonstrate that the *pilI* background mutation causes biofilm and twitching defects in the *lon*- strain. More specifically, we show that *pilI* expression in the *lon*- fully recovers biofilm formation and partially the twitching motility. We have made a new figure (new Fig. 6) to add these important data as well as a more detailed description the *pilI* mutation to our paper.

5. While the authors mentioned that the mechanistic basis of Lon's effects on these processes remains poorly understood in Discussion part, this manuscript does not sufficiently address the underlying molecular mechanisms of Lon protease activity. To provide deeper mechanistic insights, the authors should conduct additional experiments including analysis of substrate degron sequences, interaction

assay with Lon-accessory proteins, and substrate degradation assays using various protease mutant strains

We agree that our studies opens up many new interesting questions. With the newly validated Lon substrates as well as the long list of promising Lon substrate candidates, it will be interesting to address important research questions regarding Lon degrons, specific regulators and Lon's role in the broader proteostasis network. However, we think that most of these questions will require extensive experimentation and will thus exceed the scope of this paper, in which we clarify the role of Lon on pathogenicity-related phenotypes. That said, we have done a preliminary analysis of C-terminal and N-terminal sequences to see if we identify common features pointing to consensus degron sequences. However, this analysis did not reveal a striking pattern; therefore, more investigation will be needed to thoroughly characterize the degrons of the newly identified substrate proteins.

6. The manuscript does not explain the mechanism by which SulA regulates motility in P. aeruginosa. If experimental evidence is not available, the authors should propose a testable hypothesis to explain this regulatory relationship.

SulA is a direct inhibitor of cell division and its accumulation in the *lon-* results in cell filamentation. We believe that cell filamentation reduces soft agar motility for the following two reasons: 1) *Pseudomonas* is monotrichous, *i.e.* it possess a single flagellum at one of its cell poles. Given that cell division-inhibited cells can become multiple times as long as normally sized cells, they are expected to be moved by this single flagellum less efficiently than small cells. 2) The large size of cell-division inhibited cells is also expected to hamper their penetration through the soft agar, further contributing to a reduction in motility.

The reviewer raised a good point that our hypothesis regarding the relationship between cell size and motility should be tested more directly. To do so, we blocked cell division in a SulA-independent way by addition of the antibiotic piperacillin that is known to specifically block cell division. Consistent with our hypothesis, we found that piperacillin-induced cell division inhibition results in severely reduced motility, which we attribute to the increased cell size of piperacillin-treated cells. We have added the data to Fig. 5 and included a more detailed explanation of how SulA, cell division, cell size and motility are linked.

Reviewer #2: Ideally, the role of the pilI background mutation in the biofilm and twitching motility defects of the lon- should be directly tested by recreating the lon disruption in a clean PAO1 background. Alternatively, perhaps an existing pilI mutant could be tested for these phenotypes to see if the lon- strain is phenocopied with respect to biofilm formation and twitching motility? It would be interesting to know whether other lon- strains with reported biofilm defects also have type IV pilus mutations in their backgrounds, but this is outside the scope of this manuscript. However, highlighting this observation here may motivate other researchers to check the backgrounds of their lon- strains.

To directly test if the *pilI* mutation accounts for the biofilm and twitching phenotypes of the *lon-* mutant, we tested if expression of wild-type *pilI* restores biofilm formation and twitching motility in the *lon-* strain. As shown in the new Fig. 6, this was indeed the case and we have added the data and new text to describe these data in the manuscript. See also previous comments.

Part III – Minor Issues: Editorial and Data Presentation Modifications
Please use this section for editorial suggestions as well as relatively minor modifications of existing data that would enhance clarity.

Reviewer #1: Minor concerns

1. The distinction between 'significantly degraded' and 'lower stability' in lonOE strain shown in Figure 1C requires clarification, as protein degradation rate directly correlates with protein stability. This terminology should be explicitly defined and distinguished in both the main text and figure legend to avoid confusion.

We have clarified this aspect in both the main text and the figure legend.

Reviewer #2: 1. The point mutation in lon- strain should probably be listed as part of the genotype(s) in the strain table for clarity.

Done.

2. The y-axes of some panels in Fig. 1B appear mislabeled.

Thanks for pointing that out. We have corrected this and have also double-checked that all other figure labels are correct.

3. The author's statement suggests that proteases are promising drug targets (line 34). Some would argue that the AAA+ ATPase dependent proteases are very challenging drug targets. Highly conserved homologs exist in the human mitochondria for several of these proteases, making it very difficult to selectively target the bacterial proteins. Also, where small molecule inhibitors or activators of these proteins have been found, they have had poor pharmacological properties and substantial efforts to improve these properties have failed.

The reviewer is right that there might be challenges in targeting bacterial drugs by antibiotics. However, a considerable amount of research is dedicated to the investigation of bacterial proteases as antibiotic drug targets. This includes studies of how the antibiotic ADEP results in bacterial cell death by overactivating the ClpP protease and how cyclomarin and other cyclopeptides affect ClpCP in important bacterial pathogens. In light of this large body of research, we still think that our statement is valid and that new research will be needed to overcome the mentioned current limitations.

4. Ideally, raw proteomics data should be uploaded to a public repository such as PRIDE.

The proteomics data have been uploaded to PRIDE.

Figure resubmission:

While revising your submission, please upload your figure files to the Preflight Analysis and Conversion Engine (PACE) digital diagnostic tool, https://pacev2.apexcovantage.com/. PACE helps ensure that figures meet PLOS requirements. To use PACE, you must first register as a user. Registration is free. Then, login and navigate to the UPLOAD tab, where you will find detailed instructions on how to use the tool. If you encounter any issues or have any questions when using PACE, please email PLOS at figures@plos.org. Please note that Supporting Information files do not need this step. If there are other versions of figure files still present in your submission file inventory at resubmission, please replace them with the PACE-processed versions.

Reproducibility:

To enhance the reproducibility of your results, we recommend that authors of applicable studies deposit laboratory protocols in protocols.io, where a protocol can be assigned its own identifier (DOI) such that it can be cited independently in the future. Additionally, PLOS ONE offers an option to publish peer-reviewed clinical study protocols. Read more information on sharing protocols at https://plos.org/protocols?utm_medium=editorial-email&utm_source=authorletters&utm_campaign=protocols
